# Use of MALDI-TOF to identify cryopreserved mastitis pathogens collected from 2003 to 2011 that were originally identified using conventional microbiological methods

**DOI:** 10.3168/jdsc.2024-0711

**Published:** 2025-02-20

**Authors:** L. de Souza Ferreira, T. Showemimo, L.B. Juliano, Z. Rodriguez, P.L. Ruegg

**Affiliations:** Department of Large Animal Clinical Sciences, Michigan State University, East Lansing, MI 48824

## Abstract

For decades, conventional microbiological methods have been used to identify bacterial causes of bovine mastitis. Although these methods are relatively accurate for identification of important mastitis pathogens, all diagnostic tests are imperfect, and as testing technologies advance, widespread use of newer technologies may result in differences in the distribution of etiologies that are identified. As a result, historical research using conventional microbiological methods may not be comparable to results of current studies. The objective of this study was to compare agreement between the original identification of mastitis pathogens from cows enrolled in mastitis studies between 2003 and 2011 with identification of the same isolates using MALDI-TOF. Cryopreserved bacterial isolates (n = 308) that had been recovered from quarter milk samples and originally identified using conventional microbiological techniques were used. Bacterial identification was performed using MALDI-TOF. Among all isolates, 277 were able to be identified using MALDI-TOF, and the overall observed levels of agreement were 86% and 64% for identification at the genus level and genus-species level, respectively. The kappa statistic for agreement between methods at the genus level was substantial at 0.80 (95% CI: 0.78, 0.82) but dropped to 0.64 (95% CI: 0.62, 0.66) for agreement at both the genus and species level. For gram-positive isolates, agreement at both the genus level and the genus and species level was substantial. In contrast, for gram-negative isolates, the genus-level agreement was substantial, but agreement at both the genus and species levels was moderate. Our findings suggest substantial agreement between MALDI-TOF and conventional methods for determining genus-level identification, but some discrepancies occur at the species level. These results indicate that historical mastitis research using conventional microbiological methods are comparable at the genus level to current results using MALDI-TOF, but some caution should be applied when making species-level comparisons, especially for gram-negative pathogens.

## Highlights

• MALDI-TOF was used to re-identify mastitis pathogens from studies done in 2003 to 2011.
• Observed agreement between methods was >80% for the genus level but 58% to 71% at the species level.
• Cohen's kappa indicated moderate to substantial agreement in identification between methods.
• Most research based on conventional methods can be compared with results that use MALDI-TOF.

## Graphical Abstract

**Figure:**
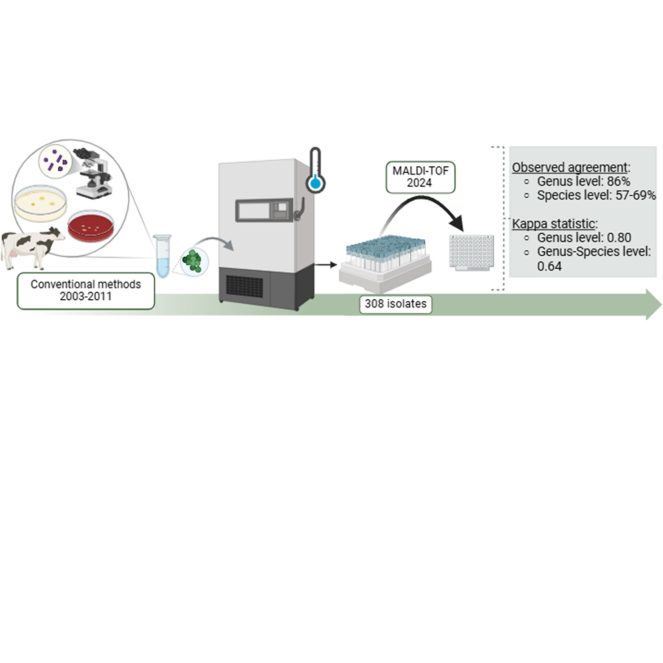
**Summary:** Cryopreserved bacterial mastitis pathogens (n = 308) previously identified by conventional methods were re-identified using matrix-assisted laser desorption/ionization time-of-flight mass spectrometry (MALDI-TOF). Findings showed substantial agreement at the genus level; however, some variation was seen at the species level. These results indicate that most results of historical research that relied on conventional microbiological methods can be integrated with results of current studies that use MALDI-TOF. Created with BioRender.com.

Bovine mastitis remains a serious concern on dairy farms, leading to substantial economic losses due to reduced milk yield, decreased milk quality, and increased antimicrobial use in adult dairy cows (Pol and Ruegg, 2007). Precise identification of bacterial pathogens responsible for IMI is essential for selecting appropriate treatment protocols and for guiding preventive management strategies (Adkins and Middleton, 2018). For decades, conventional microbiological methods have been used to identify bacterial causes of bovine mastitis. The National Mastitis Council (NMC, 1999, 2017) has established standardized guidelines that have served as the reference for bacterial identification. These methods include growth on selective agars, phenotypic appearance of colonies, Gram staining characteristics, and results of biochemical reactions. Although conventional methods are effective in identifying major mastitis pathogens, they are labor-intensive, time-consuming, and require skilled laboratory personnel.

In recent years, microbiological techniques have advanced and led to the widespread adoption of MALDI-TOF for microbial identification (Cameron et al., 2018; Jahan et al., 2021). After initial growth on traditional agars, MALDI-TOF uses high-throughput technology that identifies microorganisms by generating a peptide mass fingerprint through laser ionization. The resulting spectrum, composed of distinct peaks, is compared against a reference database of validated organisms to determine the identity of the sample (Haider et al., 2023). Several researchers have demonstrated that MALDI-TOF offers a faster, more cost-effective, and more accurate means of identifying microorganisms. Although conventional methods are relatively reliable for genus-level identification, MALDI-TOF has been shown to be more accurate for species-level identification (Nonnemann et al., 2019; Wilson et al., 2019). However, routine use of MALDI-TOF to identify mastitis pathogens has resulted in reporting of a more diverse microbiota, which may be difficult to compare with results of historical research that identified pathogens using conventional methods.

Accurate identification of mastitis pathogens is necessary to determine appropriate preventive strategies and guide decisions about interventions such as appropriate antibiotic treatments. Although there has been continued progress in controlling mastitis, decades of research are based on results of conventional microbiological methods that may have resulted in misidentification or incomplete characterization of some isolates. Variation in the identification of organisms may limit the ability to compare results of previous research based on conventional methods to results of current studies that use MALDI-TOF. The objective of this study was to compare agreement in identification of mastitis pathogens by comparing results of conventional microbiological methods used to identify bacteria from milk samples of cows enrolled in mastitis studies between 2003 and 2011 with identification of the same isolates using MALDI-TOF. We hypothesized that the methods would exhibit sufficient agreement to allow integration of results of past and current studies.

This study was a cross-sectional study with bacterial isolate as the experimental unit. The isolates were a convenience sample from the cryopreserved collection of the senior author (PLR) and originated from quarter milk samples collected from cows with clinical or subclinical mastitis, which were enrolled in trials between 2003 and 2011. The entire collection consists of about 4,700 mastitis pathogens and all isolates had been identified in the same research laboratory at the University of Wisconsin–Madison. The collection of milk samples and microbiological methods used in each study followed standard microbiological methods for mastitis pathogens used at the time (NMC, 1999) and have been previously described (Pol and Ruegg, 2007; Apparao et al., 2009; Pantoja et al., 2009; Lago et al., 2011; Oliveira et al., 2011, 2012, 2013; Pinzón-Sánchez and Ruegg, 2011; Stiglbauer et al., 2013). The organisms were selected to broadly represent the distribution of pathogens in our collection and the sample size was determined by the budget. All the isolates had been originally identified to the species level, following National Mastitis Council guidelines (NMC, 1999). The techniques were reasonably consistent for all studies and were supervised by the same trained laboratory technician for the entire period. Typical procedures included growth on blood and McConkey agar, observation of phenotypic appearance, use of secondary testing as recommended by the guidelines (such as catalase, coagulase and fermentation of mannitol or growth on selective agars), and use of miniaturized commercial biochemical tests. Isolates had been preserved with glycerin in a −80C freezer.

This study took place between June and July of 2024 in the Ruegg Laboratory in the College of Veterinary Medicine at Michigan State University (East Lansing, MI). Isolates were recovered from a −80°C freezer, streaked onto blood agar using sterile 10-µL plastic loops, and aerobically incubated at 37°C for 24 h. The following day, plates were examined for growth and purity, and bacterial identification was performed using MALDI-TOF (MALDI Biotyper 3.1 User Manual, Bruker Daltonics Inc.) with the 5.3.1 Bruker library. Briefly, a single-use, 15-cm sterile applicator stick was used to transfer a single colony onto the MALDI-TOF 96-spot target plate (Bruker Daltonics Inc., Billerica, MA). Two spots were assigned to each isolate and air-dried. Subsequently, the first spot of each isolate was overlaid with 1 µL of 70% formic acid (Sigma-Aldrich, St. Louis, MO). Once dried, the spots were overlaid with 1.0 µL of matrix (α-cyano-4-hydroxycinnamic acid). A bacterial test standard provided by the manufacturer was used in every run. The MALDI-TOF analysis was performed using a Bruker Microflex LT mass spectrometer (Bruker Daltonics, Bremen, Germany) in linear mode using Flex Control 3.4 software (Bruker Daltonics), with spectra collected across a mass range of 2,000 to 20,000 *m*/*z*. Each spectrum was generated from 240 accumulated laser shots. The resulting spectra were analyzed using MALDI Biotyper 4.1 software, with identification based on log scores. Only results with log score >1.70 were used.

Isolates that were not identified using MALDI-TOF after the first round of analysis were re-cultured, using the previously described protocols. In cases where MALDI-TOF results did not align with the identification determined by conventional microbiological methods, the isolates were subjected to an additional round of conventional microbiological testing. This involved re-culturing the isolates and re-identifying them to the genus level following NMC guidelines (NMC, 2017). The re-testing was conducted to ensure that the same isolates originally identified had been accurately recovered from the −80°C freezer.

Statistical analyses were performed using R 3.4.4 software (R Studio Inc.). Observed agreement was assessed as the percentage of samples for which the original identification and MALDI-TOF provided matching classifications. Cohen's kappa was used to assess agreement between the original identification and MALDI-TOF results, providing for agreement between methods adjusted for agreement expected by chance. Cohen's kappa values were interpreted as follows: 0.01 to 0.20 = slight agreement, 0.21 to 0.40 = fair agreement, 0.41 to 0.60 = moderate agreement, 0.61 to 0.80 = substantial agreement, and 0.81 to 1.00 = almost perfect agreement.

Of 308 cryopreserved bacterial isolates initially included, only 277 isolates (Table 1) were able to be identified using MALDI-TOF. Six isolates were excluded because they could not be confirmed (using conventional microbiological methods) to be the original isolate that was cryopreserved. After 2 attempts, 25 isolates could not be identified using the MALDI-TOF. Based on the original conventional techniques, these isolates were distributed as *Aerococcus viridans* (n = 4), *Klebsiella* spp. (n = 1), *Klebsiella oxytoca* (n = 1), *Klebsiella pneumoniae* (n = 2), *Micrococcus* spp. (n = 3), *Staphylococcus* *hyicus* (n = 2), *Staphylococcus aureus* (n = 2), *Staphylococcus* *saprophyticus* (n = 1), *Staphylococcus* *simulans* (n = 1), *Staphylococcus* *capitis* (n = 1), *Staphylococcus* *chromogenes* (n = 1), *Staphylococcus* *xylosus* (n = 1), *Streptococcus* *dysgalactiae* (n = 1), *Streptococcus* *agalactiae* (n = 1), and *Streptococcus* *suis* (n = 3). Based on the identification using MALDI-TOF, the isolates included in the final dataset were categorized as gram-positive (n = 242) and gram-negative (n = 35) organisms. The gram-positive organisms were distributed as *Staphylococcus* spp. (n = 100 NAS and 23 *Staph. aureus*), streptococci and *Streptococcus*-like organisms (**SLO**; distributed as *Streptococcus* spp. [n = 67], *Aerococcus* spp. [n = 9], *Enterococcus* spp. [n = 16], *Lactococcus* spp. [n = 23], and 4 others; Table 1).

**Table 1:** Identification of cryopreserved mastitis pathogens included in the analysis

Pathogen	Original identification using conventional methods^1^ (n)	Identification using MALDI-TOF^2^ (n)
*Aerococcus urinae*	1	0
*Aerococcus viridans*	8	9
*Corynebacterium amycolatum*	0	1
*Enterococcus*spp*.*	3	0
*Enterococcus faecalis*	5	6
*Enterococcus faecium*	4	6
*Enterobacter ludwigii*	0	4
*Enterococcus raffinosus*	0	1
*Enterococcus virans*	1	1
*Enterococcus saccharolyticus*	0	2
*Escherichia coli*	9	8
*Klebsiella*spp*.*	13	0
*Klebsiella aerogenes*	0	1
*Klebsiella oxytoca*	5	3
*Klebsiella pneumoniae*	5	16
*Kocuria*spp*.*	1	0
*Kocuria rhizophila*	0	2
*Leuconostoc citreum*	1	0
*Lactococcus garvieae*	0	3
*Lactococcus lactis*	22	20
*Mammaliicoccus sciuri*	0	3
*Micrococcus*spp*.*	2	0
*Micrococcus luteus*	0	1
*Raoultella planticola*	0	1
*Serratia*spp*.*	3	0
*Serratia marcescens*	0	1
*Serratia nematodiphila*	0	1
*Staphylococcus*spp*.*(NAS)	9	0
*Staphylococcus aureus*	23	31
*Staphylococcus auricularis*	1	1
*Staphylococcus capitis*	2	1
*Staphylococcus caprae*	1	0
*Staphylococcus chromogenes*	25	37
*Staphylococcus cohnii*	1	1
*Staphylococcus epidermidis*	12	9
*Staphylococcus equorum*	0	1
*Staphylococcus felis*	2	0
*Staphylococcus gallinarum*	0	1
*Staphylococcus haemolyticus*	7	11
*Staphylococcus. hominis*	1	0
*Staphylococcus hyicus*	12	5
*Staphylococcus lentus*	1	0
*Staphylococcus microti*	0	1
*Staphylococcus saprophyticus*	1	1
*Staphylococcus schleiferi*	1	0
*Staphylococcus sciuri*	7	0
*Staphylococcus simulans*	6	9
*Staphylococcus ureilyticus*	0	1
*Staphylococcus warneri*	0	1
*Staphylococcus xylosus*	11	9
*Streptococcus*spp*.*	5	0
*Streptococcus acidominimus*	2	0
*Streptococcus agalactiae*	10	6
*Streptococcus dysgalactiae*	25	28
*Streptococcus equinus*	1	0
*Streptococcus mitis*	2	0
*Streptococcus salivarius*	1	0
*Streptococcus suis*	1	1
*Streptococcus thermophilus*	0	1
*Streptococcus uberis*	23	31
*Streptococcus vestibularis*	1	0
Total	277	277

1 Conventional microbiological methods performed during 2003–2011.

2 MALDI-TOF performed in 2024 using cryopreserved isolates from isolates collected during 2003–2011.

A broad variety of pathogens were included (Table 1). Use of MALDI-TOF resulted in increased speciation of some *Klebsiella pneumoniae* that had been previously identified at the genus level (Table 1). Similarly, 9 organisms identified as NAS species using conventional methods were identified to the species level using MALDI-TOF (Table 1). Few differences were seen in identification of *Lactococcus* spp., although only MALDI-TOF resulted in identification of *Lactococcus garvieae* (Table 1). Accurate identification of *Staph. aureus* is important due to the infectious and chronic nature of these infections. Although 21 of 23 *Staph. aureus* that had been identified using conventional methods were re-identified as *Staph. aureus* using MALDI-TOF, use of MALDI-TOF identified additional *Staph. aureus*, most of which had been previously identified as NAS (n = 8). The conventional laboratory methods used for diagnosis of *Staph. aureus* typically included observation of hemolysis, a positive tube-coagulase test, as well as fermentation of mannitol. However, individual strains of *Staph. aureus* may vary in exhibiting these characteristics (NMC, 2017), thus resulting in errors in identification. Misidentification of NAS when using conventional methods has been well described (Sampimon et al., 2009), and MALDI-TOF identified more *Staph. chromogenes* as compared with conventional methods (Table 1). Among the *Streptococcus* spp., several *Strep. agalactiae* that had been identified using conventional methods were determined to be other *Streptococcus* spp. using MALDI-TOF.

At the genus level, observed agreement ranged from 80.0% (for organisms categorized as SLO) to 93.4% (staphylococci; Table 2). Observed agreement was less at the species level and ranged from 57.9% (gram-negative isolates) to 70.5% (for organisms categorized as SLO; Table 2). For genus-level identification of all isolates, the kappa statistic was 0.80 (95% CI: 0.78, 0.82; *P* < 0.001; n = 277), indicating substantial agreement. When assessing both genus- and species-level identification of all isolates, the kappa statistic was 0.64 (95% CI: 0.62, 0.66; *P* < 0.001; n = 241), also reflecting substantial agreement, although at a lower level.

**Table 2:** Observed agreement between MALDI-TOF and conventional methods

Item	Total, n	Observed agreement	
		Only genus, n (%)	Genus and species,^1^ n (%)
All isolates	277	237 (85.5)	155/241 (64.3)
Gram-positive^2^	242	209 (86.3)	144/222 (64.9)
*Staphylococcus* spp.^3^	123	115 (93.4)	70/115 (60.9)
SLO^4^	115	92 (80.0)	74/105 (70.5)
Gram-negative	35	28 (80.0)	11/19 (57.9)

1 Only calculated for isolates identified to the species level using both methods.

2 Includes 1 *Kocuria*, 1 *Micrococcus*, and 1 *Corynebacterium* spp.

3 Includes *Mammaliicoccus sciuri* (diagnosed as *Staphylococcus sciuri* in 2003–2011).

4 Includes *Streptococcus*, *Aerococcus*, *Enterococcus*, and *Lactococcus* spp.

For gram-positive isolates (n = 242), genus-level agreement was substantial (kappa = 0.79, 95% CI: 0.70, 0.87; *P* < 0.001), indicating a high degree of consistency between MALDI-TOF and conventional methods. At the genus and species level, agreement remained substantial but kappa dropped to 0.63 (95% CI: 0.57, 0.67; *P* < 0.001), suggesting that although genus identification is reliable, there is more variability at the species level. In contrast, for gram-negative samples (n = 35), genus-level agreement was also substantial (kappa = 0.65, 95% CI: 0.56, 0.72; *P* < 0.001), but genus- and species-level agreement were moderate (kappa = 0.57, 95% CI: 0.19, 0.85; *P* < 0.001), indicating more variability and notable discrepancies in species identification for gram-negative bacteria.

Identification of mastitis pathogens using MALDI-TOF is considered reliable. Initially developed and widely used in human clinical microbiology, MALDI-TOF has proven effective for rapid and accurate identification of a wide range of bacterial species (Haider et al., 2023). In veterinary medicine, it has been validated as an accurate secondary test (Adkins and Middleton, 2018; Nonnemann et al., 2019; Haider et al., 2023). Our study differs from earlier work in that we aimed to compare historical research results that used conventional methods with current results that use MALDI-TOF. For all isolates analyzed, we observed strong agreement between the 2 methods at the genus level (86%), which aligns with Wilson et al. (2019), who reported a 94% genus-level agreement when comparing MALDI-TOF with biochemical methods in 181 milk isolates. These findings suggest that the conventional methods were reliable for genus-level identification, as they demonstrated considerable concordance with MALDI-TOF.

Evaluations of MALDI-TOF have consistently demonstrated high accuracy in bacterial identification for both gram-positive and gram-negative bacteria. For instance, Cameron et al. (2018) highlighted the effectiveness of MALDI-TOF in identifying NAS, a group difficult to differentiate with biochemical methods, by comparing its performance against molecular techniques. Similarly, in a study involving 500 udder pathogens, 100% of isolates were accurately identified at the genus level and 93.5% at the species level when validated using PCR (Nonnemann et al., 2019). Although our purpose was not to evaluate test accuracy, we observed substantial agreement at the genus level for both gram-positive (86.3%) and gram-negative bacteria (80.0%). However, when focusing on both genus- and species-level identification, observed agreement dropped to 64.9% and 57.9% for gram-positive and gram-negative bacteria, respectively. Although conventional techniques have long served as the foundation of microbiological diagnostics and are well established as reliable for genus-level identification, available data highlight their limitations, particularly in distinguishing pathogens at the species level (Adkins and Middleton, 2018). In contrast, MALDI-TOF has superior performance in this area (Wilson et al., 2019), which aligns with our results suggesting that some of the original species-level identifications were incorrect. It is important to acknowledge that errors may have arisen from both the conventional techniques and potential limitations in the MALDI-TOF databases. Neither MALDI-TOF nor conventional methods are suitable to use as a gold standard for identification, and integration of past and current studies would be enhanced if future research included genomic methods.

Our data showed that although conventional microbiological techniques performed reliably at the genus level, agreement decreased at the species level. This limitation, however, may have minimal impact on comparing results of historical research to results of current studies, as most mastitis control programs are more broadly focused on characterization of pathogens as primarily contagious or environmental. However, discrepancies in identification of *Staph. aureus* should be recognized and additional studies performed to ensure accurate identification of this important pathogens. If future control programs require species-level identification, recommendations based on results of more recent studies that use MALDI-TOF are preferable. An important limitation of our study is that isolates had been collected for studies with specific objectives, and the distribution of isolates was unbalanced and not reflective of overall prevalence. Some pathogens (such as *Corynebacterium* spp. and bacilli) were rarely recovered and were not cryopreserved. This decision may have influenced our estimates of agreement at the species level. Additional research that includes more isolates to determine species specific agreement would allow for more generalized inferences.

We were unable to identify 8% (25/308) of the isolates using MALDI-TOF, and based on conventional techniques, most were NAS. Our results are consistent with Mahmmod et al. (2018), who reported that 9 of 115 bovine-associated NAS isolates remained unidentified after 2 rounds of MALDI-TOF. Similarly, Banach et al. (2016) observed that 6 of 33 NAS (from cows with subclinical mastitis) could not be identified using the same method. The inability to identify certain isolates with MALDI-TOF has been attributed to gaps in the reference databases used for matching protein spectra (Cameron et al., 2017; Nonnemann et al., 2019). Agreement may vary from other laboratories that use a custom database for mastitis studies. Additionally, factors such as bacterial growth conditions, cryopreservation duration, and sample preparation protocols can contribute to these challenges. Given that the bacterial isolates in our study were cryopreserved for 15 to 20 years, it is possible that alterations in protein expression due to prolonged storage could also explain the inability to identify some isolates using MALDI-TOF.

In conclusion, the broad level of agreement between conventional identification of isolates used in mastitis studies, supports the potential to integrate most historical research based on conventional microbiological methods with results of modern studies that use MALDI-TOF, thus providing confidence in conclusions of historical research studies.
